# PERINATAL GRIEF, EMERGENCY EVALUATION. ABOUT A CASE

**DOI:** 10.1192/j.eurpsy.2023.2402

**Published:** 2023-07-19

**Authors:** M. L. Maria Del Carmen, C. I. Irene, S. G. Alejandro

**Affiliations:** 1 Psiquiatría, Hospital General Universitario Gregorio Marañón; 2 Psiquiatría, Hospital Universitario de Torrejón; 3 Psiquiatría, Hospital Universitario Principe de Asturias, Madrid, Spain

## Abstract

**Introduction:**

Perinatal grief is the process that occurs after the loss of a baby, either during pregnancy or during the period immediately before or after childbirth (up to a year). In recent years, the increase in specific training and development of programs focused on perinatal mental health has facilitated the creation of specific action protocols. The case of a 38-year-old woman who suffers a gestational loss during the third month of pregnancy is explored. The presence of personal and family antecedents that suppose risk factors for the adequate elaboration of the duel, raise doubts about the handling of the case.

**Objectives:**

This work has several objectives, including reviewing the published literature on perinatal bereavement in an emergency situation and, on the other hand, presenting a case.

**Methods:**

A bibliographic search has been carried out in the main sources of medical information such as pubmed, uptodate as well as in national and international journals. Likewise, the knowledge and clinical experience of the team has been reviewed in order to expose its own experience in this field, defining specific interventions as well as results.

**Results:**

On evaluation, the patient was conscious and oriented to person, time, and space. Approachable and cooperative. Overall calm, although with intermittent crying. Low mood reactive to vital situation, without apathy, apathy, or anhedonia. No previous episodes of hypomania or mania. Not another major affective clinic. Fluid and coherent speech, formally well constructed without glimpse alterations in the course or content of thought. She denied sensory-perceptual alterations, without showing a listening attitude, or suspicion or any other psychotic or dissociative symptoms. He denied ideas of self-harm, death or self-harm, presenting an adequate request for help and coherent and realistic future plans. Altered biological rhythms with insomnia of three days of mixed pattern evolution. preserved appetite. Judgment of reality preserved.

The grief reaction is an experience that must be normalized after the loss of a loved one. However, given the risk factors presented by the patient, preventive management is established in the face of possible complicated perinatal grief. A new appointment is established in less than 10 days to reassess the case with the perinatal mental health team.

**Conclusions:**

Perinatal mental health is an area of knowledge that could provide assistance to mothers, fathers and family systems plunged into a crisis of perinatal grief.

Prevention in situations of possible complicated perinatal mourning is no less important than treatment when the disorder is already established.

**Disclosure of Interest:**

None Declared

